# Recent Advances in Surface-Enhanced Raman Scattering for Pathogenic Bacteria Detection: A Review

**DOI:** 10.3390/s25051370

**Published:** 2025-02-23

**Authors:** Yimai Wang, Zhiqiang Zhang, Yixiang Sun, Huimin Wu, Liqiang Luo, Yizhi Song

**Affiliations:** 1 Department of Chemistry, College of Sciences, Shanghai University, Shanghai 200444, China; 22725067wym@shu.edu.cn (Y.W.); wuwhm@shu.edu.cn (H.W.); 2 Suzhou Institute of Biomedical Engineering and Technology, Chinese Academy of Sciences, Suzhou 215163, China; zhangzq@sibet.ac.cn (Z.Z.); sunyx@sibet.ac.cn (Y.S.); 3 Division of Life Sciences and Medicine, School of Biomedical Engineering (Suzhou), University of Science and Technology of China, Suzhou 215163, China

**Keywords:** SERS, bacteria, nanoparticle, Raman label

## Abstract

Bacterial infection is one of the common infectious diseases in clinical practice, and the research on efficient detection of bacteria has attracted much attention in recent years. Currently, the traditional detection methods of bacteria are mainly based on cell culturing, microscopic examination, and molecular biology techniques, all of which have the disadvantages of complex operation and time-consuming. Surface-enhanced Raman spectroscopy (SERS) technology has shown prominent advantages in bacterial detection and identification because of the merit of high-sensitivity, fast detection and unique molecular fingerprint spectrum. This paper mainly investigates and discusses the application of SERS in bacterial detection, and systematically reviews the progress of SERS applications, including nano-enhanced dielectric materials of SERS, signal amplification of SERS labeled molecules, and the integration of SERS with microfluidic technology. Finally, the paper analyzes the challenges associated with the application of SERS in bacterial detection and offers insights into future development trends.

## 1. Introduction

Pathogenic infections are one of the main causes of human morbidity and mortality, and the types of pathogens that cause infectious diseases are very diverse, including viruses, bacteria, fungi, and other microorganisms. To enhance the efficiency of diagnosis and treatment of various diseases, clinical testing technology must be more accurate and rapid [1,2]. Traditional methods for bacterial detection, such as culture media, biochemical reactions, and serological diagnosis, are cost-effective and highly reproducible. However, these techniques have limitations of time-consuming procedures and the need for specialized expertise, which makes them inadequate for meeting current clinical demands [3].

Current new diagnostic methods established for rapid bacterial detection including real-time quantitative polymerase chain reaction (qPCR), enzyme-linked immunosorbent assays (ELISAs), next-generation sequencing (NGS), and mass spectrometry-based matrix-assisted laser desorption ionization-time-of-flight (MALDI-TOF). However, these techniques exhibit numerous shortcomings, such as the requirement for additional sample pretreatment, elevated testing expenses, lack of reusability and portability, and limited sensitivity and accuracy. Consequently, these limitations greatly hinder their application in bacterial infection detection for clinical tests [4,5,6,7]. The accurate identification of bacteria responsible for patient infections is essential to safeguarding public health. Diagnosis delays in this process can significantly jeopardize safety outcomes. Consequently, there is an urgent need to develop new methodologies that facilitate the precise and timely identification of bacteria, bridging the gap from basic laboratory practices to applications in clinical settings.

In recent years, Raman spectroscopy has gained significant attention in research and clinical settings due to its high efficiency, and minimally invasive real-time applications, alongside advancements in instrumentation and data processing techniques [8,9,10]. Raman scattering is an inherently fragile phenomenon, approximately 6 to 10 orders of magnitude less efficient than fluorescence [11]. The phenomenon of Raman scattering produced by molecules can be significantly enhanced when these molecules are positioned close to the surface of specifically designed nanostructured substrates, commonly referred to as surface-enhanced Raman spectroscopy (SERS) substrates [12]. SERS can effectively overcome the limitations associated with the low sensitivity of conventional Raman spectroscopy. This advanced technique facilitates the acquisition of spectrum information that is challenging for traditional Raman methods [13,14,15].

The review outlines the development of SERS-based methods for bacterial detection, with a focus on the development of SERS methods and their application in complex clinical settings. This review begins with an introduction to the enhancement mechanism of SERS, an in-depth discussion of the development of label-based and label-free SERS strategies, as well as the various SERS substrates and SERS labels that are currently being developed and also examines and discusses the computational analysis of SERS spectroscopy for bacterial detection utilizing advanced algorithms and multifunctional SERS platforms.

## 2. Principles of Surface-Enhanced Raman Scattering

The enhancement mechanisms of the SERS are very complex, and include the interaction between light and rough surfaces, the interaction between light and molecules, the frequency and polarization of the incident light. The enhancement factors of the SERS effect results from electromagnetic mechanism (EM) and chemical enhancement [16,17,18]. The mechanism of surface chemical enhancement is that when a molecule is adsorbed on the surface of a substrate through a chemical reaction, the adsorbed atoms on the surface and other co-adsorbed species, etc., may have certain chemical interactions with the molecule, and these factors have a direct effect on the electron density distribution of the molecule, i.e., the change in the polarizability affects its Raman intensity; the EM enhancement mechanism is that the free electrons on the rough metal surface under the photoelectric field undergo a collective oscillatory effect leading to a localized EM enhancement, which contributes to an enhanced SERS signal. The study of the SERS enhancement mechanism was elaborated in the paper by Roberto Pilot et al. [12].

### 2.1. Electromagnetic Field Enhancement Mechanism

**Figure 1:**
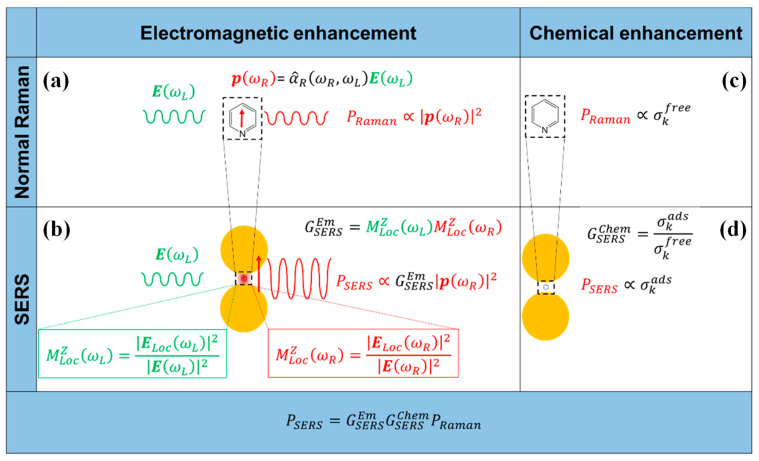
Electromagnetic enhancement. (**a**) Normal Raman. A laser radiation, with electric field *E* ($\omega_{L}$) oscillating at (angular) frequency $\omega_{L}$ impinges on a molecule, characterized by a Raman polarizability tensor ${\hat{\alpha }}_{R}$ ($\omega_{R}$, $\omega_{L}$). The laser induces a dipole oscillating at the Raman frequency (vertical red arrow, *p* ($\omega_{R}$)); the Raman power radiated by this dipole is proportional to the square modulus of the dipole itself. (**b**) SERS electromagnetic enhancement. When the molecule is placed near a plasmonic substrate, the electric field experienced by the molecule is $E_{Loc}$ ($\omega_{L}$), normally much stronger than the input laser *E* ($\omega_{L}$); this local field enhancement is quantified by $M_{Loc}^{Z}$($\omega_{L}$). Moreover, the presence of the plasmonic substrate also enhances the efficiency with which the dipole emits Raman radiation; this re-radiation enhancement is quantified by $M_{Loc}^{Z}$($\omega_{R}$). The total electromagnetic enhancement factor, within the ${\left|E\right|}^{4}$ approximation, is defined as: $G_{SERS}^{Em}=M_{Loc}^{Z}$($\omega_{L}$)$M_{Loc}^{Z}$($\omega_{R}$). Chemical enhancement. (**c**) Normal Raman. The vibrational modes of a molecule in free space are characterized by the cross-section(s) $\sigma_{k}^{free}$; (**d**) SERS chemical enhancement. The interaction with the plasmonic substrate modifies the structure of the molecule and consequently also the cross-section(s) of its modes ($\sigma_{k}^{ads}$). The chemical enhancement is quantified as $G_{SERS}^{Em}=\frac{\sigma_{k}^{ads}}{\sigma_{k}^{free}}$. Reprinted (adapted) with permission from Roberto et al. [12]. Copyright (2019), MDPI, Basel, Switzerland.

Some research works indicated that the electromagnetic field enhancement mechanism is the primary enhancement mechanism of SERS [19]. The electromagnetic and chemical mechanisms are illustrated in Figure 1. When the incident laser irradiates on a rough metal surface, the free electrons present will oscillate collectively and be coupled with the laser. This interaction generates an electromagnetic wave on the metal surface known as Surface Plasmons (SPs) [20,21,22]. Following the formation of surface excitations in proximity to nanomaterials, the resultant electric field can become several times, or even tens of times, stronger than the original electric field. This substantial enhancement positively influences the Raman signal emitted by the metal surface molecules. The excitation of the electromagnetic field is confined within a very limited region, leading to the development of localized surface plasmon excitations (LSPs).

### 2.2. Chemical Enhancement Mechanism

The chemical enhancement mechanism has a limited impact on the overall SERS enhancement process; however, it does influence the spectral pattern of the SERS effect, specifically the Raman shift and the intensity ratio of energy bands in the Raman spectrum. There are two distinct chemical effects within the chemical enhancement mechanism: non-resonant chemical effects and resonant charge transfer chemical effects. Both of these effects result in the changes of the electronic and geometric structure of the molecule [23]. However, the chemical enhancement presents certain limitations, as the molecules must either be chemically bonded or physically adsorbed to the metal surface to induce the desired enhancement effect. This phenomenon is intricately linked to various factors including the type and nanostructure of the substrate, the specific adsorption sites, the characteristics of the molecules involved, and the properties of the excitation light [19].

In the context of electromagnetic field enhancement, the SERS effect consists of two principal components, local field enhancement and re-radiation enhancement. These components collectively contribute to the overall enhancement of the electromagnetic field observed in the SERS effect.

## 3. SERS Substrate for Bacterial Detection

The substrate is crucial for enhancing SERS, as the development of multi-dimensional and high density of the “hot spots” on its surface can significantly improve the SERS signal. Additionally, the intensity of the SERS response is directly proportional to the number of hot spots present within the area under laser irradiation [24]. A considerable number of studies have been undertaken to create diverse enhancement substrates to optimize “hot spot” effects [25]. To date, a diverse array of nanomaterials has been synthesized for use as SERS substrates to enhance the Raman signals of the target molecules [26,27]. Table 1 below organizes the various types of SERS substrates developed in recent years for the identification of different species of bacteria, ranging from simple to complex substrates.

### 3.1. Single-Metal Nanoscale SERS Substrates

**Figure 2:**
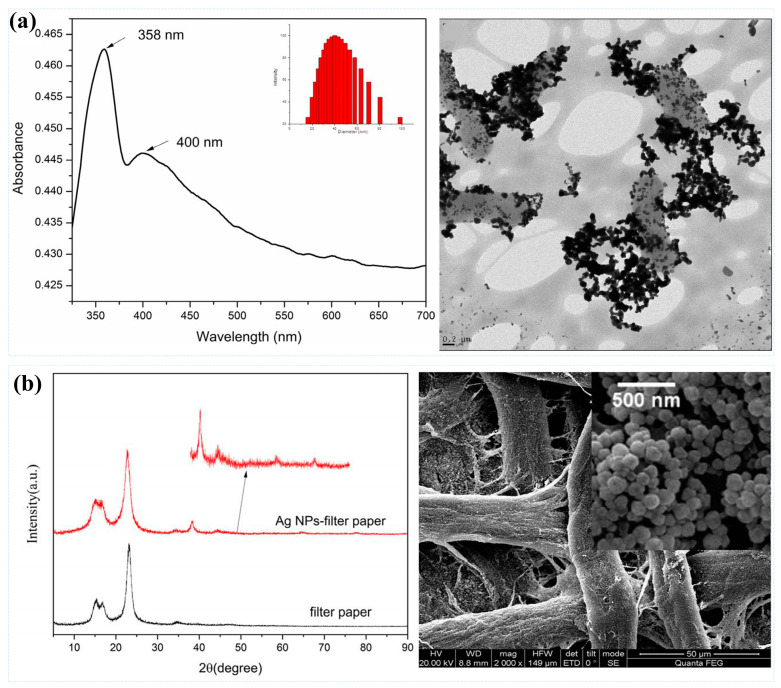
(**a**) Label-free NIR-SERS discrimination and detection of foodborne bacteria by in situ synthesis of Ag colloids: UV–visible spectrum and TEM image of Ag NPs prepared within bacteria mixture (external mode). Reprinted (adapted) with permission from Chen et al. [28]. Copyright (2015), BioMed Central. (**b**) Ag NP-filter paper-based SERS sensor coupled with multivariate analysis for rapid identification of bacteria: SEM image of the Ag NPs-filter paper substrate. Inset is the higher magnification image. Reprinted (adapted) with permission from Wang et al. [63]. Copyright (2023), Royal Society of Chemistry.

In the field of bacterial detection, gold and silver nanoparticles are the most commonly used SERS substrates [61,62]. As illustrated in Figure 2a, Chen et al. employed a label-free near-infrared surface-enhanced Raman scattering technique by in situ synthesizing silver nanoparticles within bacterial cell suspensions. This method enabled the differentiation of pathogenic bacteria, including *E. coli*, *Pseudomonas aeruginosa*, Methicillin-resistant *S. aureus* (MRSA), and *Listeria* spp., in drinking water, achieving a detection limit of 10^3^ CFU/mL [29]. Wang et al. synthesized Ag nanoparticles on filter paper by silver mirror reaction, as shown in Figure 2b, the surface of the filter paper was covered by uniformly dense silver NPs with an average particle size of about 120 nm, by which a large number of “hot spots” were able to form between neighboring Ag NPs, thus providing a high-activity and reliable SERS response [63].

### 3.2. Composited Nanoscale SERS Substrates

**Figure 3:**
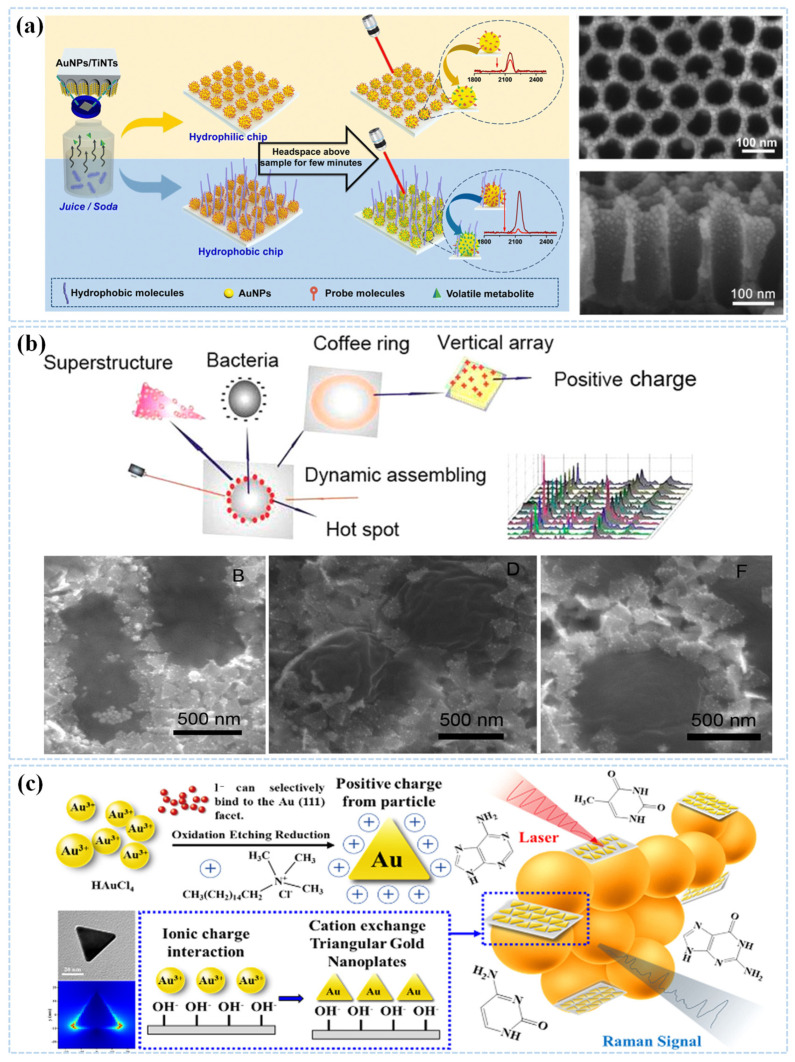
(**a**) Schematic of hydrophobic interaction assisted SERS sensing of *E. coli* metabolites and SEM images of AuNPs/TiNTs: top view and side view. Reprinted (adapted) with permission from Li et al. [70]. Copyright (2023), Royal Society of Chemistry. (**b**) Schematic illustration of the one-step assembling and detection of bacteria via plasmonic bifacial TAuNP/AuNSs superstructures on a Au@Ag nanorod columnar array; SEM image of *L. monocytogenes*, *S. xylosus* and *E. faecium* bacteria adhered to SH-PEG-NH2 functionalized bifacial TAuNP-AuNSs superstructures on a nanorod columnar array. Reprinted (adapted) with permission from Li et al. [35]. Copyright (2016), American Chemical Society. (**c**) Schematic illustration of the one-step assembling and detection of bacteria via plasmonic bifacial TAuNP/AuNSs superstructures on a Au@Ag nanorod columnar array. Reprinted (adapted) with permission from Chiu et al. [36]. Copyright (2022) Royal Society of Chemistry.

The research on nanocomposites have contributed to the development of SERS in recent years, and the SERS substrate has gradually evolved from simple gold and silver nanomaterials to composite nanomaterials [64,65,66]. Sivanesan et al. used a simple electrodeposition technique to produce a silver-gold nanobimetallic SERS substrate, which can be used for the detection of a wide range of bacteria. On the other hand, metals can be composited with non-metallic materials to form SERS substrates [67,68]. Zhang et al. combined highly transparent and mechanically robust cellulose nanofiber (CNF) biomaterials with gold nanorods to form multifunctional porous membranes for dual-mode SERS detection of small molecules and cells. The nanoporous nature of the nanofiber membranes allows for effective molecular filtration and pre-enrichment of analytes, further improved the SERS performance [69]. Li et al. designed a nanotube-based SERS substrate for the detection of methyl disulfide (DMDS), a metabolite of pathogenic bacteria, as shown in Figure 3a, where gold nanoparticles were decorated on TiO_2_ nanotubes (Au NPs/TiNTs), which were then modified with a hydrophobic monolayer and a Raman probe, phenyl acetonitrile (PA), to form a SERS composite substrate [70].

Qiu et al. designed an auxiliary plasmonic superstructure of SH-PEG-NH_2_-triangular Au nanoplates-Au nanospheres (Tau NPs-Au NSs), as shown in Figure 3b, and this double-sided assembled structure further provides a large number of “hot spots” for the enhancement of SERS. As a result, negatively charged bacteria can be efficiently trapped in the Tau NPs-Au NSs superstructure at the top of the columnar array of Au@Ag nanorods, and the dry and wet critical SERS states based on the hybridized nano-assemblies can be combined with the electromagnetic (EM) SERS effect and the photothermal temperature gradient (PTG) SERS effect for sensitive direct detection of the *S. xylosus*, *L. monocytogenes*, and *E. faecium* [35].

Chen et al. successfully synthesized Tau NPs using a one-step rapid method, stabilizing the reduction on the surface of 2D nano clay through oxidative etching, as illustrated in Figure 3c. They also controlled the edge length of the Tau NPs within the range of 2 to 30 nm by varying the concentrations of the reducing agent and NaOH [71].

## 4. SERS Technology for the Detection of Bacteria

**Figure 4:**
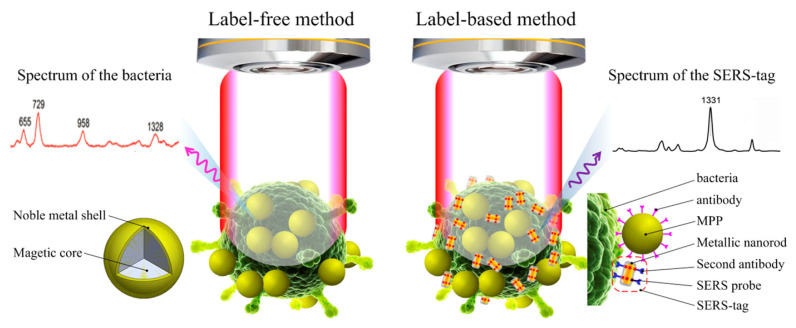
Schematic diagram of the principles of label-free and label-based SERS methods for bacterial detection. Reprinted (adapted) with permission from Wang et al. [77]. Copyright (2019), American Institute of Physics.

Because of the differences in the structure and biomolecular composition of different species of bacteria, Raman spectroscopy is specialized in identifying bacteria type and the state of bacteria because of the unique spectral fingerprints of the bacteria [72]. The detection methods for pathogenic bacteria can generally be classified into two categories: label-free and label-based detection, as illustrated in Figure 4.

The label-free detection utilizes the molecular characteristics, cellular composition, and physiological information derived from the SERS profiles of bacteria to distinguish the different types of live and dead bacteria [73,74]. For example, significant differences between *S. aureus* and *E. coli* can be detected between 1128 cm^−1^ and 1388 cm^−1^ [75]. On the other hand, the labeling detection is an indirect measurement technique in which Raman tags and target recognition components are directly affixed to the surface of metal nanoparticles. The Raman signal is generated through specific binding to the target bacteria. The intensity of the Raman signal is correlated with the quantity of bacteria being tested [76].

### 4.1. Label-Free SERS Profile of the Bacterial Pathogen

**Figure 5:**
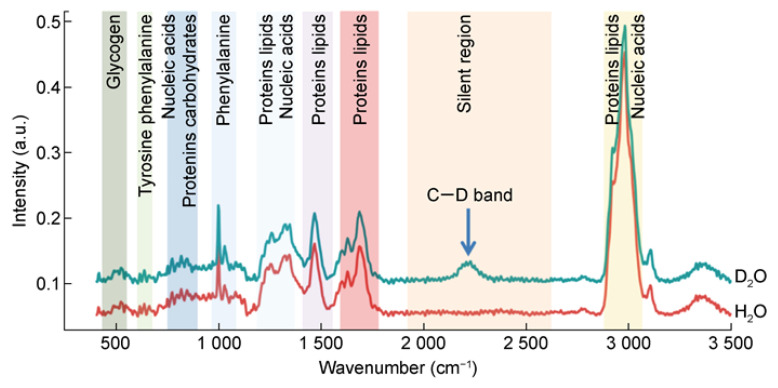
Raman spectra of *Escherichia coli* strains. Reprinted (adapted) with permission from Stöckel et al. [80]. Copyright (2015), John Wiley and Sons.

Label-free detection does not require the binding of bacteria with Raman tags, the SERS signal of the strain to be tested is obtained by directly binding the bacteria to the SERS substrate, and different Raman peaks appear for different bacteria due to the differences in the cell walls [78,79]. The Raman spectra of bacteria contain information about the different biomolecules in bacteria, as shown in Figure 5, which represent the vibrational modes of different molecules including DNA/RNA, proteins, lipids, and carbohydrates. Each pathogen has its specific chemical composition, so this information is referred to as a unique “Raman fingerprint” that identifies the pathogen [80]. Recent research primarily integrates label-free detection methods with algorithms designed to interpret SERS signals [81,82]. Furthermore, immunization techniques have been combined with SERS approaches, facilitating the label-free detection of bacteria. To date, various bacterial types have been identified by analyzing SERS signals [83].

#### 4.1.1. Label-Free SERS Binding Algorithm for Bacterial Detection

**Figure 6:**
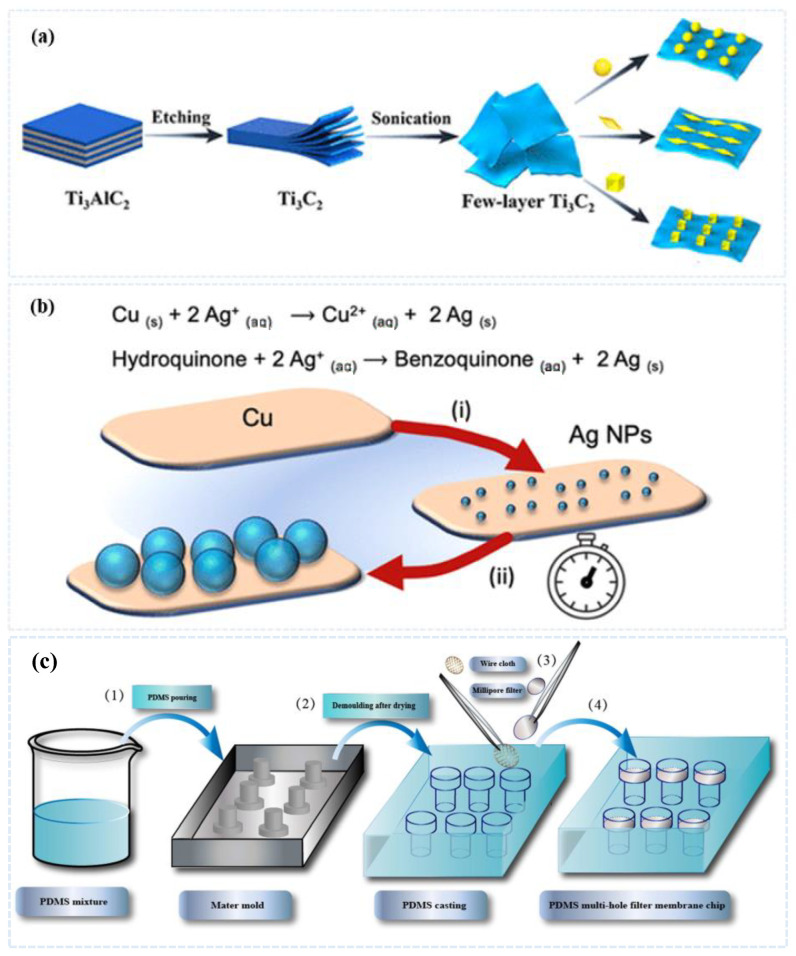
(**a**) Identification of bacterial pathogens at genus and species levels through a combination of Raman spectrometry: schematic of the fabrication process of Ti_3_C_2_-Au nanomaterial substrate. Reprinted (adapted) with permission from Jiang et al. [84]. Copyright (2023), American Chemical Society. (**b**) Cu/Ag nanoparticle-based SERS Substrates for label-free bacterial detection: schematic representation of Cu/Ag nanoparticle formation by (**i**) transient nucleation of Ag NPs and (**ii**) seed-mediated particle growth of Ag NPs nuclei in the presence of hydroquinone. Reprinted (adapted) with permission from Beyene et al. [85]. Copyright (2022), American Chemical Society. (**c**) Preparation of an Ag NPs@ Polydimethylsiloxane (PDMS) multi-hole filter membrane chip for the rapid identification of food-borne pathogens by SERS. Reprinted (adapted) with permission from Zhu et al. [86]. Copyright (2022), Elsevier.

Jiang et al. successfully combined Au and Ti_3_C_2_ by electrostatic self-assembly of nanosheets with three different morphologies, as shown in Figure 6a, and the Ti_3_C_2_-Au nanosheet layer achieved label-free and sensitive detection of *E. coli* and *S. aureus*, with a detection limit of 10^3^ CFU/mL [84]. In addition, electrochemical methods can be combined, as shown in Figure 6b. Beyene et al. reported an combination of an electrocoupling substitution reaction with a seed-mediated particle growth method for the in situ synthesis of silver nanoparticles on copper foils (Cu/Ag NPs), which synthesized a SERS substrate with strong SERS hot spot effect and greatly improved the efficiency of SERS [85].

Alia Colniță and colleagues conducted a study employing ten Raman spectra of *L. monocytogenes* for principal component analysis (PCA) discrimination. In the context of the a priori SERS methodology, a total of 145 SERS spectra of *S. Typhimurium* and 62 SERS spectra of *L. monocytogenes* were assessed within the spectral range of 600–1600 cm^−1^ for PCA analysis. The research also analyzed 83 SERS spectra of *L. Typhimurium* and 11 SERS spectra of *L. monocytogenes*, analyzed using the in situ-synthesized silver nanoparticles (Ag NPs). This findings indicated a successful differentiation between the spectra of *L. Typhimurium* and *L. monocytogenes* [87].

Wang et al. conducted an analysis of a substantial number of 30 bacterial species isolated directly from clinical samples. They visualized the average SERS spectra and examined the characteristic peaks of each spectrum. Additionally, they applied convolutional neural network (CNN) deep learning algorithms for pathogen analysis at both the genus and species levels, which is much more effective than those derived from three traditional machine learning algorithms. The CNN model achieved a remarkable classification accuracy of 99.80% at the genus level and 98.37% at the species level, and the 5-fold cross-validation results also exceeding 98% [83].

Ciloglu et al. used principal component analysis (PCA), hierarchical cluster analysis (HCA), and various supervised classification algorithms for the *S. aureus* and *L. pneumophila*, in which SERS profiles were differentiated, and these traditional classification methods showed excellent classification performance with 97.8% accuracy [88].

Recent advancements in microarray substrates have significantly accelerated the development of SERS for the characterization of bacteria. Zhu et al. created a microporous PDMS membrane chip and combined it with silver nanoparticles synthesized via the ascorbic acid redox method to serve as SERS substrate. This setup was utilized for the detection of *S. aureus*, *S. typhimurium*, and *C. perfringens*. By analyzing the differences in SERS peaks, an effective and appropriate sample preparation method was devised for collecting microorganisms on SERS substrates. As illustrated in Figure 6c, this method integrated major component analysis and stepwise linear discriminant analysis [86].

#### 4.1.2. Bacterial Detection of Label-Free SERS-Binding Biological Ligands

Nanoenzymes are nanomaterials with catalytic activity similar to that of natural enzymes that can catalyze certain organic dyes into SERS-active molecules, thus highly catalytic and stable nano enzymes offer great promise for the development of simple, stable, and highly sensitive label-free SERS assays.

**Figure 7:**
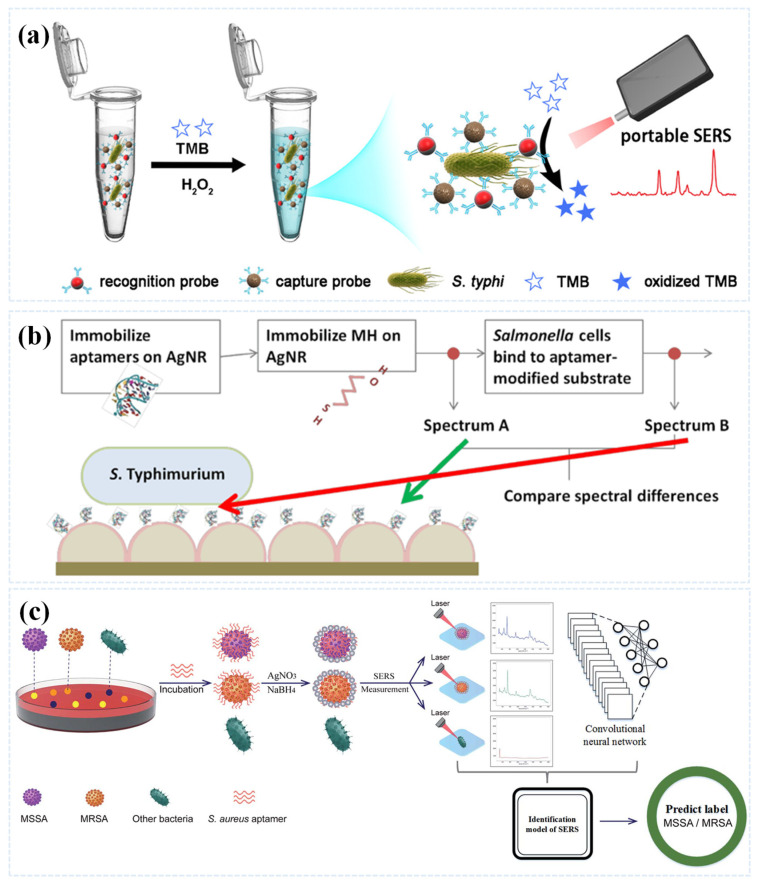
(**a**) Schematic diagram of the bifunctional Au@Pt NPs *S. typhimurium* detection process based on nucleophile nano enzymes and immunomagnetic beads. Reprinted (adapted) with permission from Li et al. [89]. Copyright (2023), American Chemical Society. (**b**) Rapid SERS identification of methicillin-susceptible and methicillin-resistant *S. aureus* via aptamer recognition and deep learning: schematic representation of rapid label-free SERS detection of MSSA and MRSA based on aptamer-guided Ag NPs formation and CNN classification. Reprinted (adapted) with permission from Wang et al. [90]. Copyright (2021), Royal Society of Chemistry. (**c**) Schematic diagram of the digital SERS chip, SERS mapping of *E. coli* at different concentrations and linear discriminant analysis of SERS signal of *E. coli*. Reprinted (adapted) with permission from Wen et al. [91]. Copyright (2024), American Chemical Society.

Li et al. utilized bifunctional Au@Pt NPs to construct a signal amplification label-free SERS assay for the highly sensitive detection of *S. typhimurium*. Firstly, the team synthesized gold nanoparticles by seed-mediated method, then synthesized bifunctional Au@Pt NPs by chemical synthesis method, and finally constructed sandwich immuno-detection system by functionalizing bifunctional Au@Pt core–shell nano-enzymes with homogeneous ultra-thin shells with antibodies against *S. Typhimurium*, and combining them with antibody-coupled magnetic beads, as shown in Figure 7a. This SERS strategy avoided Raman marker immobilization on the substrate and achieves label-free detection of *S. Typhi* [89].

Label-free SERS can also be performed by affinity recognition such as antigen–antibody interaction [92]. In addition to antibodies, nucleic acid aptamers have been proposed as a new generation of recognition units in biosensing, which are single-stranded DNA or RNA oligonucleotides produced through an iterative in vitro selection process, with a certain degree of fitness to the bacteria to be tested [93,94].

In 2021, Wang et al. developed an aptamer-guided Ag NP-enhanced and label-free SERS method for CNN classification as shown in Figure 7b, which can rapidly and accurately identify methicillin-sensitive *S. aureus* (MSSA) and methicillin-resistant *S. aureus* (MRSA), 30 strains of MSSA and 100 strains of MRSA were used to establish a CNN classification model with an accuracy of 100% [90]. In recent years, the development of microfluidic technology has improved the efficiency of SERS to detect bacteria, and some scholars have combined chip technology with SERS substrate to better isolate the detected bacteria. In 2024, Wen et al. proposed a novel digital SERS chip. As depicted in Figure 7c, the chip mainly consisted of an inverted pyramid microcavity array, a microchannel cover plate, and a multilayer nanoparticle substrate positioned at the bottom of the microcavities. The microcavity array and microchannel cover plate worked together to achieve digital discretization of the sample solution, while the inverted pyramid microcavities and multilayer nanoparticle substrate enabled highly sensitive in situ SERS detection [91].

### 4.2. Bacterial Detection of Label-Based SERS

When multiple bacteria are present in complex environments, bacterial detection becomes more complicated and more difficult to distinguish bacteria from the complex samples, making it difficult for label-free SERS assays to achieve selective quantitative detection of pathogenic bacteria [25]. To address this problem, various SERS tags have been developed for tag-based bacterial SERS detection, and SERS tags usually consist of SERS-active nanoparticles (Ag NPs or Au NPs) and SERS labeling molecules [95,96,97]. Currently, the main Raman-labeled molecules are shown in Table 2, different Raman-labeled molecules have different characteristic peaks and different detection limits.

#### 4.2.1. Bacterial Detection of Label-Based SERS Conjugated Immunity

**Figure 8:**
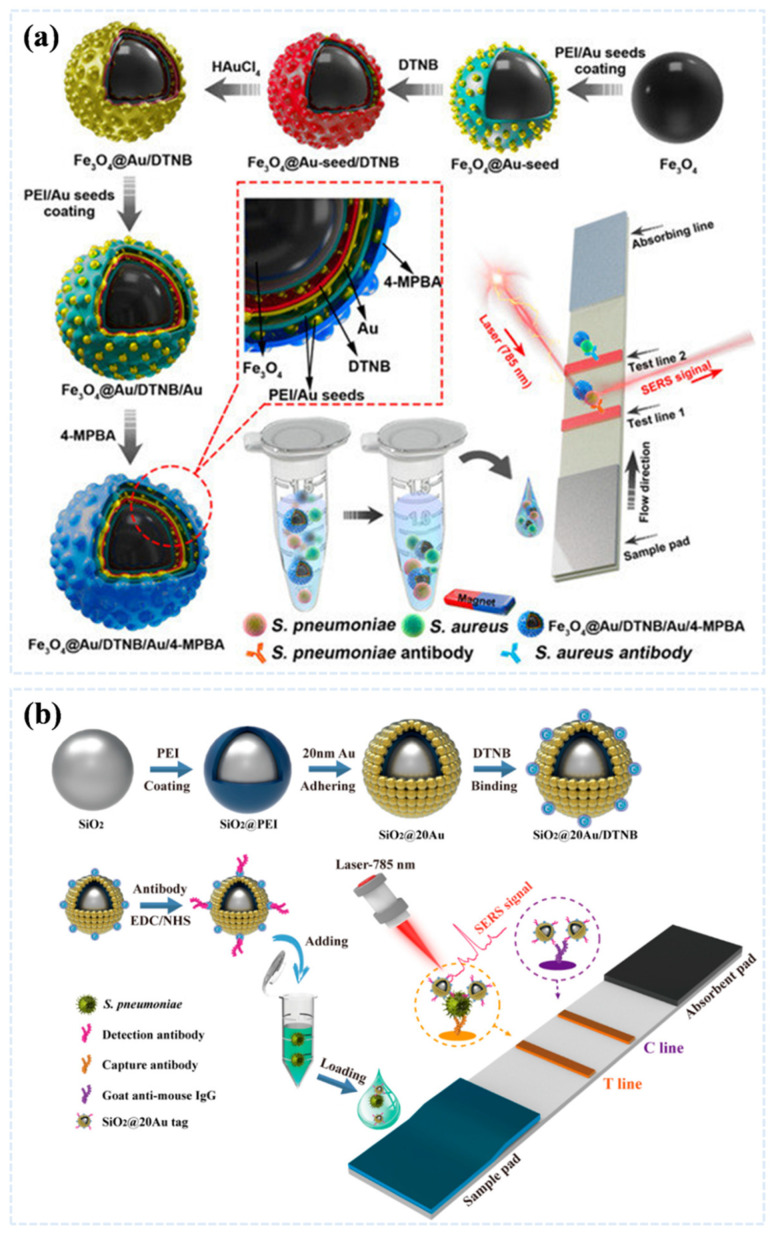
(**a**) Magnetic SERS strip based on 4-mercaptophenylboronic acid-modified Fe_3_O_4_@Au for active capture and simultaneous detection of respiratory bacteria. Reprinted (adapted) with permission from Li et al. [55]. Copyright (2023), MDPI, Basel, Switzerland. (**b**) Schematic of the preparation of SiO_2_@20Au nanocomposite, preparation of antibody-conjugated SiO_2_@20Au tag, and SiO_2_@20Au-based SERS-ICA for the quantitative of *S. pneumoniae.* Reprinted (adapted) with permission from Shen et al. [103]. Copyright (2023), MDPI, Basel, Switzerland.

Lateral flow immunoassay (LFA) has become the most mature on-site diagnostic technique after undergoing decades of development due to its advantages, such as rapidity, simple operation, low cost, and portability [102]. In 2023, Li et al. designed a rapid, ultrasensitive, and quantitative LFA strip for the simultaneous detection of the respiratory bacteria *S. aureus* and *S. pneumoniae* as shown in Figure 8a. The assay was designed by combining magnetite with the SERS tag Fe_3_O_4_@Au/DTNB. The recognition element 4-mercapto phenylboronic acid (4-MPBA) Fe_3_O_4_@Au/DTNB-mediated magnetic enrichment and 4-MPBA mediated universal capture capability improved the detection sensitivity, with detection limits as low as 8 and 13 CFU/mL for *S. aureus* and *S. pneumoniae*, respectively, which were lower than the detection line of the colloidal gold method. The Fe_3_O_4_@Au/DTNB/Au/4-MPBA-LFA also showed good reproducibility, excellent specificity, and high recovery in sputum samples, indicating its potential application in the detection of respiratory bacterial samples [103].

Shen et al. developed a straightforward and efficient immunochromatography (ICA)-based SERS label utilizing SiO_2_@Au NPs for the sensitive and quantitative detection of *S. pneumoniae*, as illustrated in Figure 8b, dense 20 nm gold nanoparticles were electrostatically adsorbed onto 20 nm SiO_2_ NPs, producing a core with remarkable stability and SERS activity. This core was then coupled with DTNB Raman-labeled molecules to enhance signal amplification. The introduction of the ICA immunoassay ensured high sensitivity and accuracy in the detection process. The limit of detection of the proposed SERS-ICA method for *S. pneumoniae* reached to 46 CFU/mL, demonstrating a sensitivity that is 100 times greater than that of traditional colorimetric ICA methods relying on gold nanoparticles [77].

#### 4.2.2. Bacterial Detection of Label-Based SERS-Binding Biological Ligands

Recognition components such as aptamers, and small molecule ligands can be coupled to markers for the identification of specific bacteria [34]. These SERS tags enable isolation and enrichment by identifying and specifically capturing desired analytes in complex sample matrices, highly selective nucleic acid aptamers that can be used in place of antibodies for complex and rapid identification of bacteria, and detection of different pathogens by synthesizing a large number of SERS tags with different Raman-labeled molecules and specific recognition elements [84,104].

**Figure 9:**
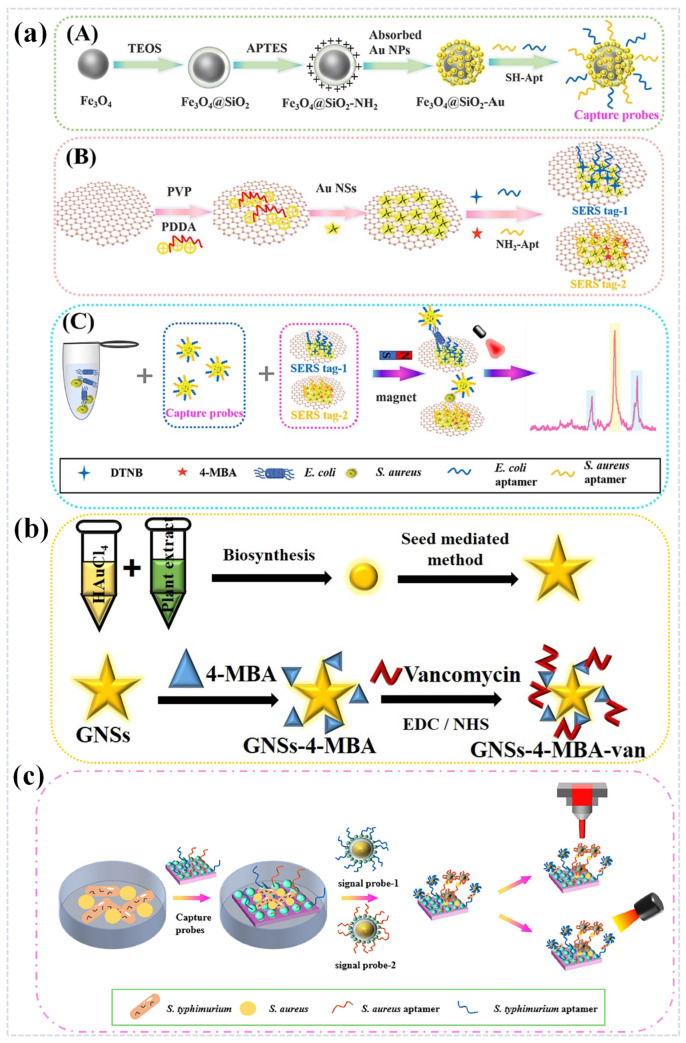
(**a**) Schematic diagram of preparation of (**A**) Apt-Fe_3_O_4_@SiO_2_-Au magnetic capture probes, (**B**) aptamers conjugated SERS tags and (**C**) dual-enhanced strategy for simultaneously detecting *E. coli* and *S. aureus*. Reprinted (adapted) with permission from Zhao et al. [105]. Copyright (2023), Elsevier. (**b**) The synthesis steps of GNSs using Au seeds, and vancomycin modified GNSs (GNSs-4-MBA-van). Reprinted (adapted) with permission from Dayalan et al. [107]. Copyright (2022), John Wiley and Sons. (**c**) Schematic representation of the synthesis process for specific detection and photothermal killing of *S. typ* and *S. aureus*. Reprinted (adapted) with permission from Dai et al. [108]. Copyright (2025), Elsevier.

In 2023, Zhao et al. developed an innovative SERS sandwich strategy biosensing platform for the simultaneous detection of *E. coli* and *S. aureus*. As depicted in Figure 9a, the researchers began by preparing gold nanocomposites (NCs) containing varying amounts of gold nanocrystals and examined the impact of inter-particle gaps on SERS activity using a finite-difference time-domain method. The optimal magnetic SERS-active substrate was then functionalized with a specific nucleic acid aptamer, which acted as a capture probe. Furthermore, Raman-labeled molecular MBAs and nucleic acid aptamer-modified graphene oxide-gold nanostars (GO-Au NSs) were employed as SERS labels. To increase the number of SERS active sites, the loading density of Au NSs on GO was adjusted. Under optimal conditions, this SERS platform can detect *E. coli* and *S. aureus* simultaneously, achieving a detection limit as low as 10 CFU/mL [105].

In addition to nucleic acid aptamers, antibiotics can also be used for molecular recognition as part of a SERS tag bound to a nano substrate [106]. Dayalan et al. developed a SERS Van-sensor based on a dual-recognition strategy for the multiplexed detection of *A. baumannii* and *K. pneumoniae* in food and clinical samples, as shown in Figure 9b, where they used the new structure made of the vancomycin’s polyethyleneimine (PEI)-intercalated superparamagnetic iron oxide (SPION)-gold nanoparticles (Au NPs). Molecular target probes were first synthesized by labeling Au NPs with 4-mercaptobenzoic acid (4-MBA), and then, Au NPs-4-MBA were functionalized with vancomycin to form an Au NPs-4-MBA-Van SERS label. Based on this dual recognition strategy, highly specific, sensitive, and simultaneous detection of target bacteria was achieved, and the detection limit of this study was 10 CFU/mL [107].

In 2025, Dai et al. designed and constructed a multifunctional SERS biosensor based on sandwich structure of “capture probe/bacteria/signal probe” in order to simultaneously identify, detect and kill *S. typ* and *S. aureus*. As shown in Figure 9c, Aptamer-modified ZnO/Ag was used as a capture probe to accurately identify and capture the target bacteria in complex environments. Au@Ag-4-MPBA-Aptamer was employed as signal probe to provide the corresponding bacterial SERS “fingerprint” information. The SERS enhancement mechanism of the sandwich-structure ZnO/Ag-Au@Ag SERS substrate was discussed. The sandwich-type SERS biosensor exhibited the strong localized surface plasmon resonance (LSPR) effect and the detection limit for *S. typ* and *S. aureus* was as low as 10 CFU/mL. Furthermore, the sandwich-type SERS biosensor offered excellent photothermal conversion efficiency (54.32%), enabling photothermal killing of target bacteria when exposed to laser irradiation [108].

## 5. Machine Learning-Assisted Raman Spectroscopy and SERS

Traditional analytical methods often struggle to differentiate subtle variations among closely related pathogenic microorganisms or to discern specific signals from background interference. SERS, despite its substantial advantages and capacity to yield sophisticated analytical outcomes, encounters significant challenges in interpreting complex spectral data. Machine learning algorithms emerge as crucial computational tools, enabling the extraction of meaningful patterns and the identification of significant biomarkers from intricate Raman/SERS data [109,110].

**Figure 10:**
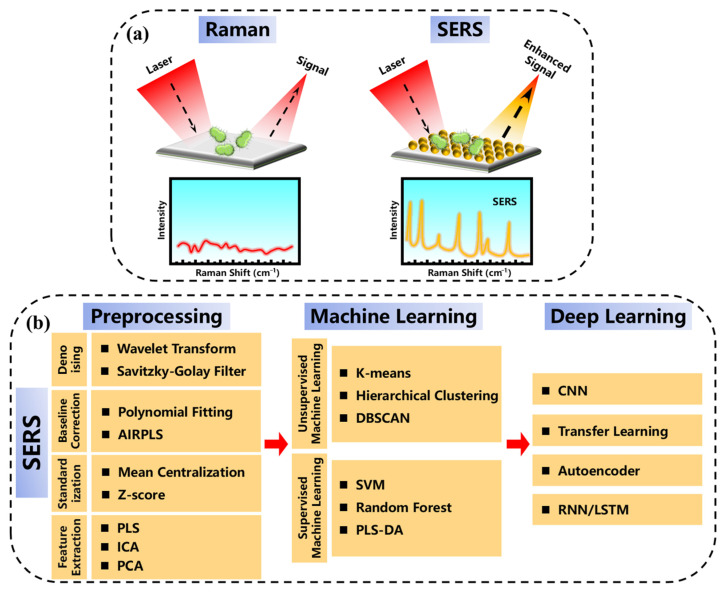
(**a**) Schematic diagram of conventional Raman and SERS for the detection of bacteria; (**b**) SERS detection preprocessing method and data analysis based on machine learning.

Figure 10a illustrates the difference between Raman and SERS for bacterial detection and the convergence of machine learning and Raman spectroscopy within the past five years has unleashed a new era for rapid, label-free bacterial pathogen detection. As shown in Figure 10b, the preprocessing includes denoising, baseline correction, standardization, and feature extraction, depending on the processing method.

### 5.1. Machine Learning Techniques for SERS Spectrum Analysis

In the context of SERS data analysis, both unsupervised and supervised machine learning approaches offer distinct advantages and are applicable to specific scenarios; therefore, selecting the most suitable algorithm based on research objectives remains a critical consideration for scholars in bacterial detection studies.

#### 5.1.1. Unsupervised Machine Learning

Unsupervised learning enables direct analysis of the intrinsic spectral structure without requiring labeled datasets, thereby facilitating preliminary species identification and structural characterization of pathogenic microorganisms. Representative unsupervised learning techniques, including K-means clustering, hierarchical clustering analysis, and DBSCAN (Density-Based Spatial Clustering of Applications with Noise), offer significant advantages in microbial analysis. These methods not only enable the identification of previously unrecognized bacterial subgroups but also provide dimensionality reduction for streamlined data interpretation and serve as valuable tools for hypothesis generation in fundamental research [111,112].

#### 5.1.2. Supervised Machine Learning

Supervised machine learning algorithms establish predictive models through a two-phase process: initially training on a curated dataset comprising labeled input features and corresponding known outputs, followed by deployment of the trained model to predict outcomes for novel input data. This methodological framework offers robust theoretical guarantees and rigorous predictive capabilities. The predictive accuracy of supervised learning models is fundamentally dependent on the quality and representativeness of the training dataset, as the model’s performance is intrinsically linked to the statistical relationship between the learning set and new data [113]. Supervised machine learning algorithms, including support vector machine (SVM), random forest, partial least squares discrimination analysis (PLS-DA) and others, offer a complementary approach, often focusing on extracting handcrafted features from spectral data [114].

### 5.2. Deep Learning Techniques for SERS Spectrum Analysis

Deep learning encompasses a powerful suite of learning techniques that leverage multilayered neural networks to uncover complex relationships within data. In deep learning technology, CNN are a good choice for processing spectral signals and exhibit excellent performance in identifying complex spectral patterns. Wang et al. proposed a deep learning model combined with SERS spectra to distinguish methicillin-resistant and susceptible *S. aureus*. The CNN model was able to import a large amount of data for automatic feature extraction and parameter fine-tuning, and was able to achieve a 100% differentiation rate between the two bacteria [90]. In addition to this, there are other deep learning technologies, such as Autoencoder for dimensionality reduction and transfer learning for small samples.

## 6. Current Challenges

Future research in the field of surface-enhanced Raman spectroscopy (SERS) will place a strong emphasis on understanding the accuracy and inherent limitations of this innovative technique. A significant hurdle facing researchers is the non-reproducibility of SERS results, which poses challenges in the reliable diagnosis of bacterial pathogens within complex, real-world environments such as blood, urine, and sputum. Typically, these biological samples require the dilution or careful removal of interfering biomolecules that can obscure accurate readings.

SERS substrates, while promising, often fall prey to interference from a variety of substances present in these biological matrices. This can lead to issues such as non-specific adsorption, cross-reactivity with other molecules, and challenges associated with targeting bacteria at ultra-trace levels. To combat the faint Raman signals produced by bacteria, especially when present in low concentrations, it is imperative to develop SERS substrates that are both highly active and homogeneous, capable of providing substantial signal enhancements.

In addition to improving the substrates, there is a crucial need to optimize the preprocessing of SERS data. This involves establishing tailored methods specifically designed to address and mitigate interferences encountered in diverse contexts, thereby enhancing the overall reproducibility of the technique. As it stands, recent documentation reveals that the precise components of the bacterial cell wall that elicit the SERS signal remain largely unidentified, highlighting a key area for future investigation. By tackling these multifaceted challenges, the field can accelerate its progress in bacterial detection methodologies, ultimately encouraging more researchers to delve into the complexities and potentials of this intriguing area of study.

## 7. Conclusions

In this paper, we have examined the development of diverse SERS substrates, ranging from monometallic nanoparticles to complex 3D structures, emphasizing their role in signal enhancement and target capture. The strategic use of SERS labels, including Raman reporters, antibodies, aptamers, and even antibiotics, has further improved sensitivity and specificity. These studies demonstrate that SERS-based detection methods could rapidly detect low concentrations of bacteria with high sensitivity as well as specificity. There are still some challenges remaining in SERS-based pathogens detection, such as the lack of standardized methods, non-reusable substrate materials, interference from environmental factors and expensive portable SERS equipment. Moreover, we identify inherent limitations in label-dependent SERS nanoparticles, primarily due to the complex synthesis and biofunctionalization processes that require multidisciplinary expertise in nanotechnology, surface chemistry, and molecular biology for effective implementation. Future research should focus on addressing these challenges, exploring novel SERS-active materials, and developing robust, portable SERS-based diagnostic devices for point-of-care applications. The continued development of SERS promises to transform bacterial diagnostics and contribute significantly to combating infectious diseases.

## Figures and Tables

**Table 1:** Different types of SERS substrates for bacterial detection applications.

SERS Substrate	Bacterial Species	LOD (CFU/mL)	Ref.
Ag NPs	*E. coli*, *P. aeruginosa*, *Listeria*, MRSA	10^3^	[28]
Ag–Au NPs	*E. coli*, *S. enterica*, *S. epidermidis*, *B. megaterium*	10^4^	[29]
Au@Ag NPs-Mussel Shell	*E. coli*, *P. aeruginosa*, *S. aureus*	5 × 10^3^	[30]
POEGMA/Ag NPs	*S. aureus*	8	[31]
Ag NR array-VAN	*E. coli*, *S. enterica*, *S. epidermidis*	10^2^	[32]
Au-PLLA mat	*S. aureus*	-	[33]
Au@Ag NPs-3D Nanoassembly	*E. coli*	10^4^	[34]
TAuNP–AuNSs-Nanorod Array	*S. xylosus*, *L. monocytogenes*, *E. faecium*	50, 10^2^ and 10^2^	[35]
Au: Ag FS nylon	*S. aureus*, *P. aeruginosa*, *S. Typhimurium*	10^3^	[36]
Ag/ZnO/rGO	*E. coli*	10^4^	[37]
Au@Ag-GO-4-MPBA	*E. coli*, *S. aureus*, *P. aeruginosa*	10	[38]
G@Ag NPs@Si	*E. coli*, *S. aureus*	10^8^	[39]
Fe_3_O_4_@Au-Van MNPs	*E. coli*, *S. aureus*	20–50	[40]
Apt-Fe_3_O_4_@Au MNPs	*S. aureus*	10	[41]
PATP-AuMNPs	*S. aureus*	10	[42]
IgG@Fe_3_O_4_/Au@Ag@PDA	*S. aureus*, *E. coli*, *S. dysenteriae*, *P. aeruginosa*, *K. pneumonia*	10	[43]
Ta@Ag porous array	*E. coli*	10^2^	[44]
Ag@PDA@SiO_2_ Nanofibrous-membranes	*E. coli*, *S. aureus*	10^4^	[45]
NCs-ThMo array	*Brucella*	10^4^	[46]
Au@DTNB@PA/Fe_3_O_4_@Au-Apt MNPs	*E. coli*, *L. monocytogenes*, *S. Typhimurium*	10, 10, and 25	[47]
Ti_3_C_2_Tx-AuNPs	*E. coli*, *S. aureus*	3 × 10^5^	[48]
ACEK-SERS chip	*E. coli*, *S. aureus*	3	[49]
Au@Ag NPs Stuffed-Nanopancakes	*S. aureus*, *E. coli*, *P. aeruginosa*	7	[50]
ConA-Fe_3_O_4_@SiO_2_ NPs/Apt-Au NNPs	*S. aureus*	50–10^4^	[51]
Fe_3_O_4_-Au@RGO nanocomposite	*S. aureus*	10^4^	[52]
GO@Au/Ag-based SERS-LFA	*E. coli*, *S. Typhimurium*, *S. aureus*, *L. monocytogenes*	9	[53]
Fe_3_O_4_@Au@Ag@apt nanocomposite	*S. aureus*, *E. coli*, *P. aeruginosa*, *S. Typhimurium*, *L. monocytogenes*	10	[54]
Fe_3_O_4_@Au/DTNB/Au/ 4-MPBA-LFA	*S. aureus*, *S. pneumoniae*	8 and 13	[55]
AuAg@PB 4-MPBA MOF	*E. coli*, *S. aureus*	6 and 2	[56]
(ZnO@Ag)-ZnO NFs/Ag NPs-array	*S. aureus*, *E. coli*, *V. parahemolyticus*	10, 10^2^ and 10^2^	[57]
Ti_3_C_2_Tx@Au NPs@4-MBN	*E. coli*	10	[58]
MNP@AMP/Apt@Au@PBA nanocomposite	*S. aureus*, *E. coli O157:H7*	50–1600	[59]
Ti_3_C_2_Tx@Au NPs films	MRSA	10	[60]

NPs: Nanoparticles.

**Table 2:** Characteristic peaks, detection limits, and detected bacteria of Raman-labeled molecules.

Reporter	SERS Peak (cm^−1^)	LOD	Pathogens	Ref.
4-ATP	1077, 1587	10 CFU/mL	*S. aureus*	[98]
4-MBA	1085, 1592	13 CFU/mL	*S. aureus*	[99]
DTNB	1333	10 CFU/mL	*S. aureus*	[42]
4-Pyridinethiol	1013, 1578	10^3^ CFU/mL	*S. Typhimurium* *S. aureus*	[100]
R6G	1350	3 CFU/mL	*E.coli*	[101]
EV	798, 1367, 1616	30 CFU/mL	*S.typhimurium*	[36]
MB	1620	fM	*S.aureus* *A. baumannii K.pneumoniae*	[102]

## Data Availability

All cited references are listed in PubMed and Web of Science.
